# Co-selection of genetic antibiotic resistance in *Streptococcus pneumoniae* after repeated azithromycin mass drug administrations in Niger

**DOI:** 10.1128/aac.01562-25

**Published:** 2025-12-23

**Authors:** Armin Hinterwirth, Cindi Chen, Daisy Yan, Lina Zhong, Zhaoxia Zhou, YuHeng Liu, Jeremy Keenan, Kieran S. O'Brien, Elodie Lebas, Travis C. Porco, Thomas M. Lietman, Thuy Doan

**Affiliations:** 1 Francis I Proctor Foundation, University of California 115281 , San Francisco, California, USA; 2 Department of Ophthalmology, University of California 336868, San Francisco, California, USA; 3 Department of Epidemiology and Biostatistics, University of California 166607, San Francisco, California, USA; 4 Institute for Global Health Sciences, University of California 254285, San Francisco, California, USA; Columbia University Irving Medical Center, New York, New York, USA

**Keywords:** childhood mortality, mass drug distribution, AMR, MORDOR, Niger

## Abstract

The spread of antibiotic resistance is a major concern in the setting of azithromycin mass drug distribution for childhood mortality. We performed long-read whole-genome sequencing and phenotypic resistance analysis on *Streptococcus pneumoniae* isolated from the nasopharynx of Nigerien children from communities treated with either six twice-yearly azithromycin distributions or placebo. Here, we showed that co-selection for macrolide, tetracycline, and trimethoprim-sulfamethoxazole genetic resistance was detected with repeated azithromycin mass drug distributions.

**CLINICAL TRIALS:**

This study is registered with ClinicalTrials.gov as NCT02047981.

## INTRODUCTION

Biannual azithromycin mass drug administration (MDA) improves childhood mortality but also selects for antibiotic resistance (AMR) (1, 2). Of concern, previous work suggested that prolonged broad-spectrum antibiotic MDA might result in the co-selection of multiple resistance genes affecting both macrolide and non-macrolide resistance (3). However, mechanistic understanding was limited. MORDOR (*Macrolides Oraux pour Réduire les Décès avec un Oeil sur la Résistance*) was a cluster-randomized trial that evaluated the effects of MDA on childhood mortality in sub-Saharan countries. Thirty communities in the Dosso region of Niger were randomly selected for AMR monitoring and randomized 1:1 to receive either azithromycin (≥20 mg/kg) or placebo every 6 months for 3 years among children aged 1 to 59 months. In this secondary analysis using nasopharyngeal samples collected in the MORDOR study, we performed both antibiotic sensitivity tests and long-read whole-genome sequencing (WGS) on cultured *Streptococcus pneumoniae* to understand AMR selection when MDA is repeatedly distributed for childhood mortality.

Of the 130 nasopharyngeal isolates from 130 children subjected to long-read WGS, 122 isolates were confirmed as *Streptococcus pneumoniae* and were included in the analysis (Fig. S1 and Methods in Supplementary Material). Communities and characteristics of children are shown in Table S1. The mean (standard deviation) treatment coverage was 82.7% (8.7%) for the placebo arm and 78.4% (10.7%) for the azithromycin arm over the three-year study duration (Fig. S1)

**Fig 1:**
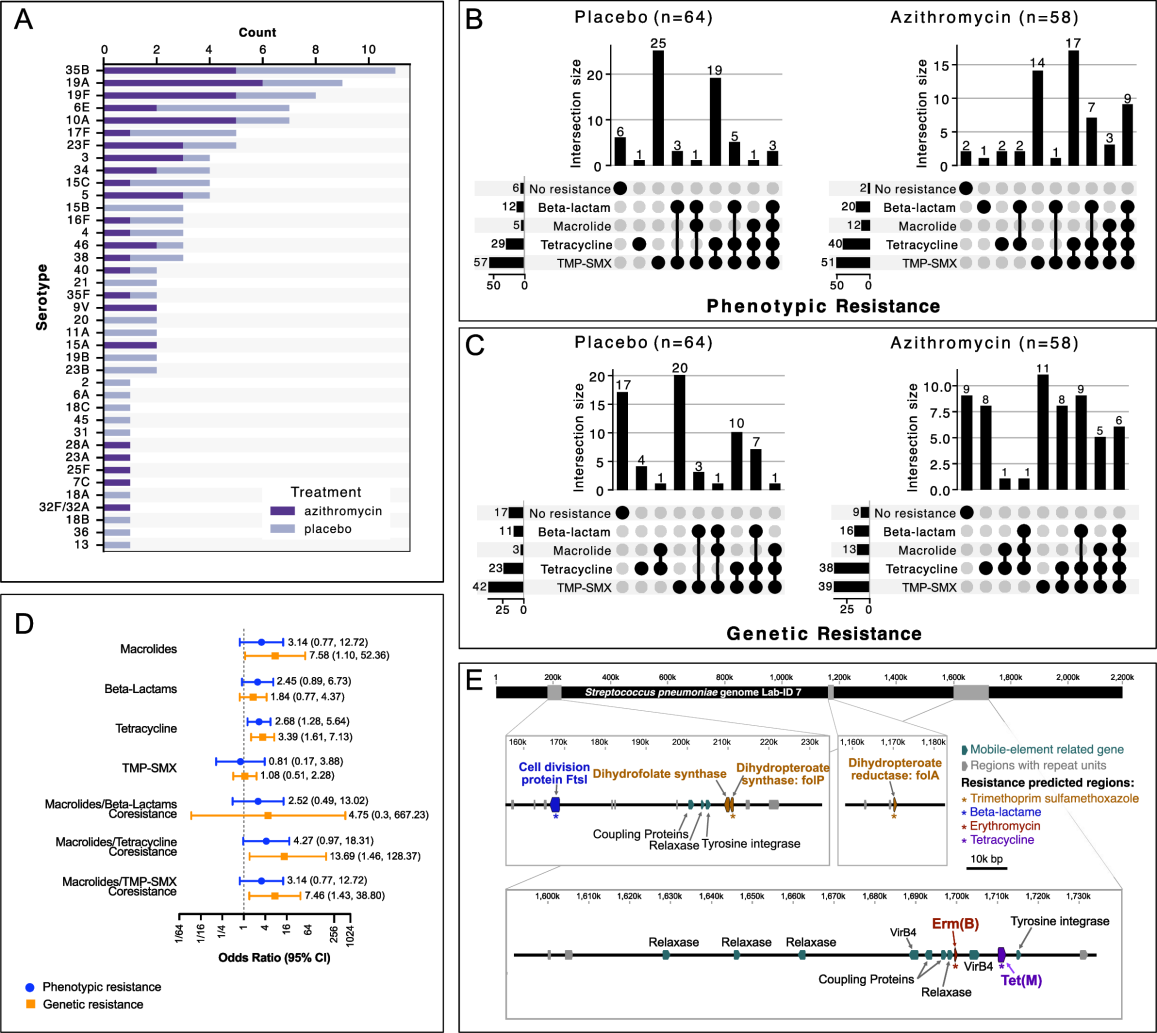
Nasopharyngeal pneumococcal antibiotic resistance isolated from preschool children after six biannual azithromycin treatments. (**A**) Stacked bar graph of WGS-based *S. pneumoniae* serotypes in preschool children at 36-month time point. UpSet plots showing the distribution and overlap of antibiotic resistance across four resistance classes (beta-lactam, macrolide, tetracycline, and trimethoprim-sulfamethoxazole) stratified by the treatment group for phenotypic (**B**) and genetic resistance (**C**). Each vertical bar represents a unique combination of resistances, with filled circles below indicating which resistance classes are present in that combination. The horizontal bars on the left show the total number of isolates resistant to each antibiotic class. Phenotypic resistance was determined based on Clinical and Laboratory Standards Institute (CLSI) breakpoints. Genetic resistance was predicted based on the AdaBoost algorithm using BV-BRC bioinformatics analysis of WGS data. (**D**) Odds ratios of antibiotic resistance at the class level in the azithromycin-treated group compared to the placebo-treated group with associated 95% confidence interval (95% CI). Blue circles and associated 95% CI indicate phenotypic resistance, whereas orange squares and associated 95% CI represent genetic resistance. *P*-values were computed from a generalized linear mixed-effects model, with cluster as a random effect. (**E**) *S. pneumoniae* was isolated from a preschool child in a community that underwent six twice-yearly azithromycin mass drug distributions. WGS indicates that this isolate contains genes conferring resistance to four classes of antibiotics. The locations of those genes are marked along a linearized *S. pneumoniae* genome. The insets provide the relative positions for AMR-associated genes. *folP* is located on an integrative mobilizable element (IME). *ermB* and *tetM* resistance genes are located on an integrative and conjugative element (ICE).

WGS-based pneumococcal serotyping was successfully performed on 115/122 samples. The top three serotypes were 35B, 19A, and 19F (Fig. 1A). The phenotypic AMR profiles based on CLSI standard methods for the 122 *S*. *pneumoniae* isolates are shown in Fig. 1B. The overall macrolide resistance burden was lowest among the four antibiotic classes. The concordance between laboratory phenotypic resistance and genetic resistance for macrolides was 0.82 (95% CI: 0.68–0.97; *P* < 0.001) (Table S2). Overall agreement between phenotypic resistance and genetic resistance for beta-lactam, tetracycline, and trimethoprim-sulfamethoxazole (TMP-SMX) ranged from 0.32 to 0.87, indicating fair to substantial agreement (Table S2).

*S. pneumoniae* isolated from children in communities treated with 3 years of azithromycin MDA had nominally higher odds of macrolide resistance than pneumococcal isolates from children in communities treated with placebo (phenotypic resistance OR = 3.14; 95% CI: 0.77–12.72; *P* = 0.11; genetic resistance OR = 7.58; 95% CI: 1.10–52.36; *P* = 0.04) (Fig. 1D). Azithromycin MDA also increased the odds for tetracycline resistance (phenotypic resistance OR = 2.68; 95% CI: 1.28–5.64; *P* = 0.01; genetic resistance OR = 3.39; 95% CI: 1.61–7.13; *P* < 0.01), but not for resistance to lactams or TMP-SMX (Fig. 1D).

Sixteen isolates had macrolide and non-macrolide co-resistance (Fig. 1B and C). Of those, 15 isolates had both genetic resistance to macrolides and tetracyclines (two isolates in the placebo-treated arm and 13 isolates in the azithromycin-treated arm; OR = 13.69; 95% CI: 1.46–128.37; *P*=0.02) (Fig. 1C through E). The genes associated with macrolide and tetracycline resistance in these isolates were *mefA*/*ermB* and *tetM*, respectively. Evidence for macrolide co-resistance with TMP-SMX was also observed with azithromycin MDA (two isolates in the placebo-treated arm and 11 isolates in the azithromycin-treated arm; OR = 7.46; 95% CI: 1.43–38.80; *P* = 0.02). All 13 isolates had mutations in the *folA* and *folP* genes conferring TMP-SMX resistance. Furthermore, these 13 isolates also carried *tetM*, indicating multi-drug resistance (MDR) as defined by resistance to >3 antibiotic classes. 85% (11/13) of the isolates were 10A, 15C, 35B, and 38, and not covered by PCV13, which was implemented in Niger and the Dosso region since 2014. Across all 16 isolates with co-resistance, integrative and conjugative elements (ICE) screening of the genomes identified either complete or partial ICE components belonging to the Tn916, Tn1549, Tn5252, and TnGBS2. MDA was not notably associated with co-resistance to macrolides and beta-lactams (OR = 4.75; 95% CI: 0.03–667.23; *P* = 0.54) (Fig. 1D).

While there is an extensive body of literature demonstrating that azithromycin MDA, whether for trachoma or childhood mortality, selects for macrolide resistance, the evidence for co-selection is less definitive (3, 4). In this analysis using pneumococcal isolates from an RCT in Niger, we demonstrated the co-selection of tetracycline and TMP-SMX resistance. Furthermore, our results suggest that the mechanism underlying these co-selections is mediated by mobile genetic elements (MGEs).

Horizontal gene transfer with MGEs, such as ICE and plasmids, is a well-documented mechanism in which MDR is spread among *Streptococcus pneumoniae* (5). Among the 122 isolates sequenced, 16 isolates had genetic evidence of co-resistance for macrolides and non-macrolide antibiotic classes, and 14 were resistant to three or four antibiotic classes. Genes conferring resistance to tetracyclines and macrolides were generally found on the same IME/ICE. While this finding provides mechanistic insight, its public health importance is limited as neither macrolides nor tetracyclines are first-line antibiotics for *S. pneumoniae*. Genes conferring beta-lactam or TMP-SMX resistance were found on separate MGEs. Thus, it appears that while selection for *mefA*/*ermB* in the setting of azithromycin MDA would also select for *tetM*, co-selection for beta-lactam and TMP-SMX-associated resistance genes is likely to have occurred independently. Indeed, beta-lactam and TMP-SMX resistance in *S. pneumoniae* in sub-Saharan Africa is common (6).

The genetic co-resistance findings in pneumococci isolated from the nasopharynx of preschool children in communities that received six rounds of azithromycin MDA, using long-read WGS sequencing, are consistent with the co-resistance findings in the gut of children from the same communities using bulk, short-read DNA-seq (3). The isolation of *S. pneumoniae* prior to WGS allows for clarification of mechanisms for co-selection of AMR that is lacking with bulk DNA-seq. This, however, is not without a tradeoff for scale, as bulk DNA-seq can interrogate resistance determinants across all pathogens to provide quantifiable changes in resistance for all antibiotic classes. From a surveillance standpoint, genetic analysis appears to be a viable approach as genetic changes precede phenotypic changes.

This study has multiple limitations. The trial had to operate under adverse conditions, including insecurity and historic flooding, and therefore MDA coverage was not consistently high across the distributions. Thus, the AMR results presented in this study may not be representative of populations outside the study setting or those receiving MDA coverage beyond the range reported here. While azithromycin MDA is likely the predominant driver of AMR detected, the identification of pneumococcal isolates harboring macrolide and TMP-SMX or tetracycline co-resistance in the placebo group suggests that other factors contribute to background co-resistance in these communities. Possible explanations, although speculative, include the widespread use of antibiotics in livestock or the routine access to antibiotics among children living in proximity to healthcare centers (7, 8). Therefore, programmatic considerations of AMR should take into account environmental variables and both documented (e.g., seasonal malaria chemoprevention) and undocumented (e.g., livestock) use of antibiotics in the relevant communities. This sub-analysis is limited by the small number of pneumococcal isolates analyzed, as reflected in the large confidence intervals for many of the comparisons performed. More importantly, these isolates were obtained from healthy children. Therefore, the correlation between MDR and virulence is unclear. Future studies would benefit from the characterization of clinical isolates from sick children who seek care at health post centers, focusing on serotypes, virulence, and vaccines, as well as the general use of antibiotics outside and within programmatic MDA. Ultimately, it would be important to understand the relationship between MDR and overall survival in the setting of MDA for childhood mortality.

In summary, this study detected the co-selection of genetic elements that confer macrolide and non-macrolide resistance after repeated azithromycin MDA for childhood mortality. MGEs appear to be an important mechanism for the accumulation of multiple AMR genes in pneumococci isolated from the nasopharynx of healthy children. Reassuringly, resistance to beta-lactams, an essential therapeutic for *S. pneumoniae*, was not associated with azithromycin MDA for childhood mortality. Additional studies are needed to determine the persistence and virulence potential of these resistance strains.

## Data Availability

Consensus whole-genome sequences are available at DOI: https://doi.org/10.5061/dryad.vt4b8gv5w. Requests for additional information are subject to approval by the MORDOR Study Group and must comply with all applicable legal and regulatory requirements. The request will be addressed within 120 days, and a data transfer agreement may be required.

## SUPPLEMENTAL MATERIAL

Supplemental material aac.01562-25-s0001.pdf Methods; Tables S1 and S2; Fig. S1.

ASM does not own the copyrights to Supplemental Material that may be linked to, or accessed through, an article. The authors have granted ASM a non-exclusive, world-wide license to publish the Supplemental Material files. Please contact the corresponding author directly for reuse.
