# Supplementary material for: Co-selection of genetic antibiotic resistance in Streptococcus pneumoniae after repeated azithromycin mass drug administrations in Niger
Source: Antimicrob Agents Chemother. 2025 Dec 23;70(2):e01562-25. doi: 10.1128/aac.01562-25 (PMC12888896; doi:10.1128/aac.01562-25)

SUPPLEMENTARY MATERIALS

METHODS

Trial Methods: The MORDOR (*Macrolides Oraux pour Réduire les Décès avec un Oeil sur la Résistance*) studies received ethical approval from the University of California, San Francisco (UCSF) Committee for Human Research and the Ethical Committee of the Niger Ministry of Health and were undertaken in accordance with the Declaration of Helsinki. Informed consent was obtained from guardians of children.

MORDOR was a cluster-randomized trial that evaluated the effects of MDA on childhood mortality in Niger, Malawi, and Tanzania for 2 years (1). In Niger, the study was prespecified to continue for another year if the study showed statistical significance (2). 30 communities in the Dosso region of Niger were randomly selected for enrollment in the smaller morbidity study to monitor AMR. Communities were randomized in a 1:1 ratio to either azithromycin (≥20 mg/kg) or placebo every 6 months for 3 years to all children aged 1 to 59 months. All personnel were masked.

Laboratory Methods and Bioinformatics: Previously, nasopharyngeal samples collected from a random sample of 40 children per community were processed for resistance sensitivities at ARUP, a CLIA-certified lab. Phenotypic results were published (3). Viable colonies from a subset of samples were sent back to UCSF for biobanking. Here, those isolated pneumococcal colonies were randomly chosen to be re-cultured and subjected to long-read WGS using the SMRTbell Prep Kit 3.0 and sequenced on the PacBio Revio platform (Pacific Biosciences of California). PacBio HiFi reads were *de novo* assembled using Canu (version 2.2) with the '-

pacbio-hifi' option and evaluated for quality using BUSCO (version 5.7.1) (4, 5). Assemblies achieving a completeness score >90% underwent a 'Comprehensive Genome Analysis' using BV-BRC online tools (accessed April 2025) (6). Because phenotypic resistance can be the result of a single gene or variations of multiple genes, machine learning algorithm AdaBoost classifiers were used to classify genetic resistance at the class-level based on long-read WGS (7). BV-BRC-annotated genomes were subsequently screened for mobile elements utilizing ICEscreen (version 1.3.3) (8). Serotypes were predicted using PfaSTer (GitHub commit 1406b83) and PneumoKITy (version 1) (9, 10).

Statistical Methods: To assess the relationship between AMR at the class-level and treatment groups, mixed-effects binary logistic regression was performed, where community of origin was treated as the random-effect and treatment group was treated as the fixed-effect. This was performed for both genetic resistance, as the outcome, determined by using BV-BRC online tools, and phenotypic resistance, as the outcome, determined previously by ARUP (7). Concordance between genetic and phenotypic resistance was assessed using Cohen's Kappa, a measure of inter-rater reliability for a categorical classification, which was calculated using the *Kappa()* function from the *vcd* package. All statistical analyses were performed using R version 4.3.1. Mixed-effects models were fit using the *glmer()* function from the *lme4* package (11). Given that antibiotic resistance was an adverse outcome, no corrections for multiple comparisons were performed. P-values less than a 2-sided alpha of 0.05 were considered notable.

REFERENCES (Methods only)

1. Keenan JD, Bailey RL, West SK, Arzika AM, Hart J, Weaver J, Kalua K, Mrango Z, Ray KJ, Cook C, Lebas E, O'Brien KS, Emerson PM, Porco TC, Lietman TM. 2018.

Azithromycin to Reduce Childhood Mortality in Sub-Saharan Africa. N Engl J Med 378:1583-1592.
2. Keenan JD, Arzika AM, Maliki R, Boubacar N, Elh Adamou S, Moussa Ali M, Cook C, Lebas E, Lin Y, Ray KJ, O'Brien KS, Doan T, Oldenburg CE, Callahan EK, Emerson PM, Porco TC, Lietman TM. 2019. Longer-Term Assessment of Azithromycin for Reducing Childhood Mortality in Africa. N Engl J Med 380:2207-2214.
3. Hazel A, Arzika AM, Abdou A, Lebas E, Porco TC, Maliki R, Doan T, Lietman TM, Keenan JD, Blumberg S. 2023. Temporal Trends in Phenotypic Macrolide and Nonmacrolide Resistance for Streptococcus pneumoniae Nasopharyngeal Samples Up to 36 Months after Mass Azithromycin Administration in a Cluster-Randomized Trial in Niger. Am J Trop Med Hyg 109:1107-1112.
4. Koren S, Walenz BP, Berlin K, Miller JR, Bergman NH, Phillippy AM. 2017. Canu: scalable and accurate long-read assembly via adaptive k-mer weighting and repeat separation. Genome Res 27:722-736.
5. Manni M, Berkeley MR, Seppey M, Zdobnov EM. 2021. BUSCO: Assessing Genomic Data Quality and Beyond. Curr Protoc 1:e323.
6. Olson RD, Assaf R, Brettin T, Conrad N, Cucinell C, Davis JJ, Dempsey DM, Dickerman A, Dietrich EM, Kenyon RW, Kuscuoglu M, Lefkowitz EJ, Lu J, Machi D, Macken C, Mao C, Niewiadomska A, Nguyen M, Olsen GJ, Overbeek JC, Parrello B, Parrello V, Porter JS, Pusch GD, Shukla M, Singh I, Stewart L, Tan G, Thomas C, VanOeffelen M, Vonstein V, Wallace ZS, Warren AS, Wattam AR, Xia F, Yoo H, Zhang Y, Zmasek CM, Scheuermann RH, Stevens RL. 2023. Introducing the Bacterial and Viral Bioinformatics Resource Center (BV-BRC): a resource combining PATRIC, IRD and ViPR. Nucleic Acids Res 51:D678-d689.
7. Davis JJ, Boisvert S, Brettin T, Kenyon RW, Mao C, Olson R, Overbeek R, Santerre J, Shukla M, Wattam AR, Will R, Xia F, Stevens R. 2016. Antimicrobial Resistance Prediction in PATRIC and RAST. Scientific Reports 6:27930.
8. Lao J, Lacroix T, Guédon G, Coluzzi C, Payot S, Leblond-Bourget N, Chiapello H. 2022. ICEscreen: a tool to detect Firmicute ICEs and IMEs, isolated or enclosed in composite structures. NAR Genomics and Bioinformatics 4.
9. Lee JT, Li X, Hyde C, Liberator PA, Hao L. 2023. PfaSTer: a machine learning-powered serotype caller for Streptococcus pneumoniae genomes. Microb Genom 9.
10. Sheppard CL, Manna S, Groves N, Litt DJ, Amin-Chowdhury Z, Bertran M, Ladhani S, Satzke C, Fry NK. 2022. PneumoKITy: A fast, flexible, specific, and sensitive tool for Streptococcus pneumoniae serotype screening and mixed serotype detection from genome sequence data. Microb Genom 8.
11. Bates D, Mächler M, Bolker B, Walker S. 2015. Fitting Linear Mixed-Effects Models Using lme4. Journal of Statistical Software 67:1 - 48.

Table S1: Demographics of Analyzed Participants

Variable	Azithromycin (N=58)	Placebo (N=64)	Total (N=122)	*P*-Value
**Communities, n**	14	15	29	0.84
**Children per Community**				
Mean (SD)	4.1 (0.9)	4.3 (2.1)	4.2 (1.6)	
Mean (95% CI)	4.1 (3.6, 4.7)	4.3 (3.1, 5.4)	4.2 (3.6, 4.8)	
Median (IQR)	4.0 (4.0, 4.0)	5.0 (2.5, 5.5)	4.0 (3.0, 5.0)	
**Gender**				0.16
Female	31 (53.4%)	26 (40.6%)	57 (46.7%)	
Male	27 (46.6%)	38 (59.4%)	65 (53.3%)	
**Age, months**				0.67
Mean (SD)	29.3 (17.0)	27.9 (18.2)	28.6 (17.6)	
Median (IQR)	27.5 (13.5, 41.0)	26.0 (12.0, 44.0)	27.0 (12.2, 42.8)	

Table S2: Agreement Between Phenotypic Resistance and Genetic Resistance for Macrolides and Non-Macrolide Antibiotic Classes.

Antibiotic Class	Cohen's Kappa (95% CI)
Macrolides	0.82 (0.68, 0.97)
Beta-Lactams	0.62 (0.46, 0.78)
Trimethoprim-Sulfamethoxazole	0.32 (0.16, 0.48)
Tetracycline	0.87 (0.78, 0.96)
Macrolides / Beta-Lactams	0.74 (0.53, 0.96)
Macrolides/ Tetracycline	0.82 (0.66, 0.97)
Macrolides / Trimethoprim-Sulfamethoxazole	0.70 (0.5, 0.89)

Fig. S1. Trial Profile

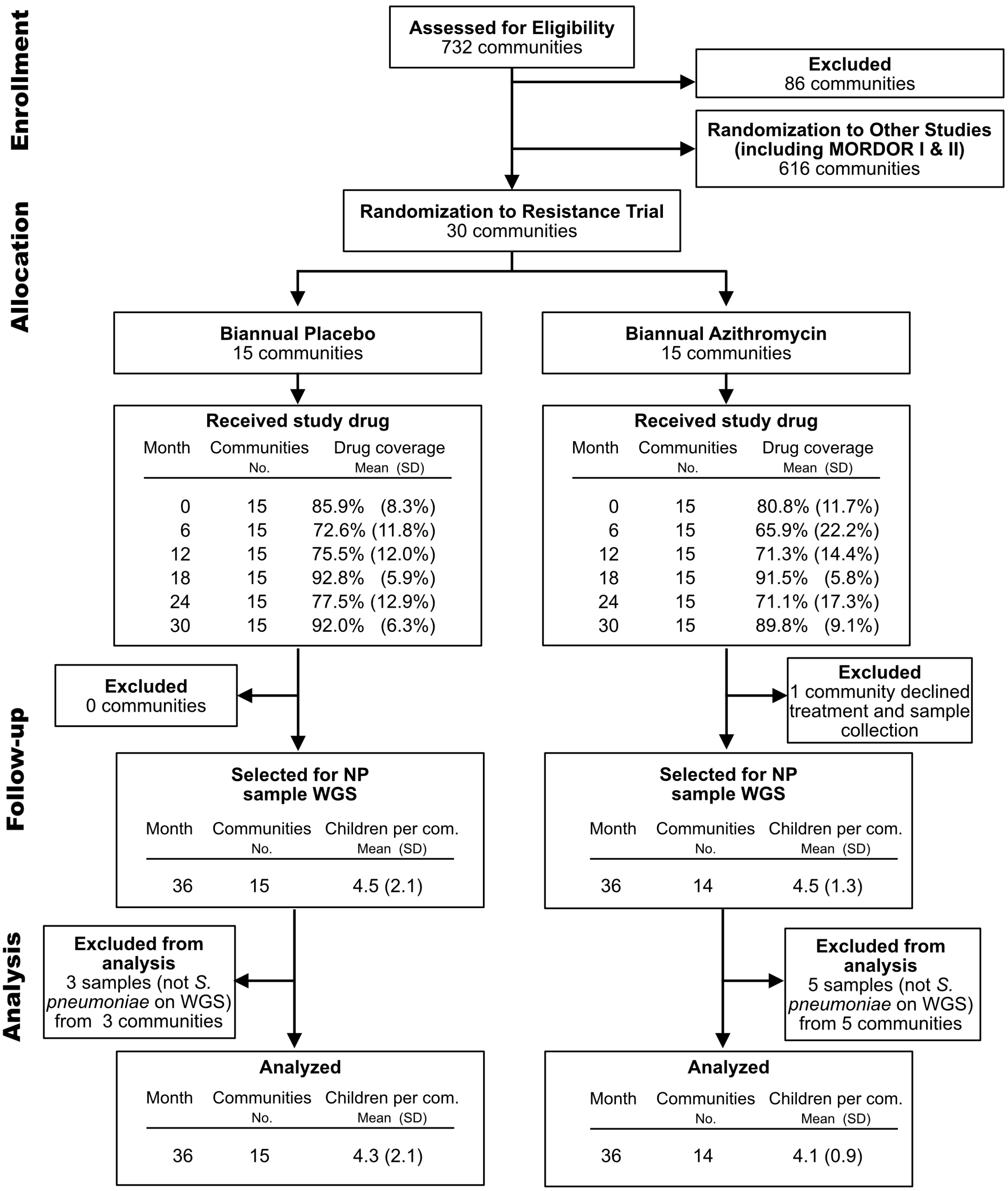

Supplement: Supplemental material — Methods; Tables S1 and S2; Fig. S1. [file aac.01562-25-s0001.pdf]
